# Supplementary material for: High Extra Virgin Olive Oil Consumption Is Linked to a Lower Prevalence of NAFLD with a Prominent Effect in Obese Subjects: Results from the MICOL Study
Source: Nutrients. 2023 Nov 4;15(21):4673. doi: 10.3390/nu15214673 (PMC10647576; doi:10.3390/nu15214673)
Supplement: Supplementary file 1 [file nutrients-15-04673-s001.zip › nutrients-2672630-supplementary.pdf]

**Table S1**		1°tertile 0-24 gr/day	2°tertile 25-37 gr/day	3°tertile >37 gr/day	p-trend
Obese					
	HDL-C	44(37;52)	46(39;54.5)	47(42;56)	0.0001
	LDL-C	123(103;142)	122(101;146)	122(101;145)	0.67
	Triglycerides	124(89;185.5)	128.5(84;175)	120(88;169)	0.48
	Glucose	105.5(99;114.5)	106(99;118)	107(99;121)	0.22
	Bilirubin	0.77(0.62;0.96)	0.77(0.65;0.97)	0.79(0.65;0.98)	0.13
	ALT	18(12;25.5)	16(12;23)	15(12;19)	0.001
	AST	12(10;14.5)	12(10;14)	11(10;13)	0.83
	GGT	13(10;18)	13.5(10;19)	14(10;18)	0.28
overweight					
	HDL-C	47(41;57)	48.5(41;58)	50(42;60)	0.03
	LDL-C	127(106;150)	123(99;143)	121(98;146)	0.03
	Triglycerides	97(67;150)	100(75;146)	101.5(73;150)	0.29
	Glucose	101(96;108)	102(96;111)	103(96;112)	0.11
	Bilirubin	0.84(0.7;1)	0.81(0.66;0.98)	0.82(0.67;1.09)	0.75
	ALT	14(11;20)	13(10;18)	13(11;18)	0.06
	AST	11(9;13)	11(9;13)	11(9;13)	0.03
	GGT	12(9;17)	12(9;16)	13(9;17)	0.07
normo weight					
	HDL-C	57(46;68)	55.5(46.5;65)	52(44;65)	0.14
	LDL-C	116(98;138)	116.5(95.5;138)	113(90;133)	0.19
	Triglycerides	75(54;109)	79.5(60;108.5)	86.5(65;121)	0.005
	Glucose	98(93;104)	99(92;104)	100(93;106)	0.13
	Bilirubin	0.81(0.65;0.96)	0.84(0.67;1.01)	0.81(0.65;1.01)	0.46
	ALT	11(9;15)	11(9;15.5)	12(9;16)	0.18
	AST	10(9;12)	10(9;12)	11(9;13)	0.0001
	GGT	9(7;12)	10(8;14)	12(8;16)	<0.0001

Metabolic profile of each BMI category by EVOO consumption tertiles. Reported statistics are: median(q1;q3)

Table S2. Schofield equations for estimating BMR from weight (kg) and height (m)

Gender /	Age (years)	BMR (kcal/day)
**Males**		
	10-17	16.2 x Weight + 137 x Height + 516
	18-29	15.0 x Weight – 10 x Height + 706
	30-59	11.5 x Weight – 2.6 x Height + 877
	60+	9.1 x Weight + 972 x Height - 834
**Females**		
	10-17	8.4 x Weight + 466 x Height + 200
	18-29	13.6 x Weight + 283 x Height + 98
	30-59	8.1 x Weight + 1.4 x Height + 844
	60+	7.9 x Weight + 458 x Height + 17.7
